# Longitudinal analysis of external quality assessment of immunoassay-based steroid hormone measurement indicates potential for improvement in standardization

**DOI:** 10.3389/fmolb.2024.1345356

**Published:** 2024-01-31

**Authors:** Laura Vierbaum, Nathalie Weiss, Patricia Kaiser, Marcel Kremser, Folker Wenzel, Mario Thevis, Ingo Schellenberg, Peter B. Luppa

**Affiliations:** ^1^ INSTAND e.V., Society for Promoting Quality Assurance in Medical Laboratories, Duesseldorf, Germany; ^2^ Faculty of Medical and Life Sciences, Furtwangen University, Villingen-Schwenningen, Germany; ^3^ Institute of Biochemistry/Center for Preventive Doping Research, German Sport University Cologne, Cologne, Germany; ^4^ Institute of Bioanalytical Sciences (IBAS), Center of Life Sciences, Anhalt University of Applied Sciences, Bernburg, Germany; ^5^ Institute of Clinical Chemistry and Pathobiochemistry, University Hospital Rechts der Isar, Technische Universität München, Munich, Germany

**Keywords:** external quality assessment (EQA), proficiency testing (PT), steroid hormones, immunoassays, accuracy, standardization, testosterone, progesterone and 17β-estradiol

## Abstract

As hormonal disorders are linked to several diseases, the accurate quantitation of steroid hormone levels in serum is crucial in order to provide patients with a reliable diagnosis. Mass spectrometry-based methods are regarded as having the highest level of specificity and sensitivity. However, immunoassays are more commonly used in routine diagnostics to measure steroid levels as they are more cost effective and straightforward to conduct. This study analyzes the external quality assessment results for the measurement of testosterone, progesterone and 17β-estradiol in serum using immunoassays between early 2020 and May 2022. As reference measurement procedures are available for the three steroid hormones, the manufacturer-specific biases were normalized to the reference measurement values. The manufacturer-specific coefficients of variation were predominantly inconspicuous, below 20% for the three hormones when outliers are disregarded, however there were large differences between the various manufacturer collectives. For some collectives, the median bias to the respective reference measurement value was repeatedly greater than ±35%, which is the acceptance limit defined by the German Medical Association. In the case of testosterone and progesterone determination, some collectives tended to consistently over- or underestimate analyte concentrations compared to the reference measurement value, however, for 17β-estradiol determination, both positive and negative biases were observed. This insufficient level of accuracy suggests that cross-reactivity continues to be a fundamental challenge when antibody detection is used to quantify steroids with a high structural similarity. Distinct improvements in standardization are required to provide accurate analysis and thus, reliable clinical interpretations. The increased accuracy of the AX immunoassay for testosterone measurement, as observed in the INSTAND EQAs between 2020 and 2022, could be the result of a recalibration of the assay and raises hope for further improvement of standardization of immunoassay-based steroid hormone analyses in the coming years.

## 1 Introduction

Hormones are biochemical messengers that play a key role in regulating the complex processes of human metabolism. Steroid hormones, such as testosterone, progesterone and 17β-estradiol, control the gender formation and maturation, as well as human reproductive processes.

Steroid hormone disorders are linked to a wide variety of health impairments, e.g., menstrual cycle disorders, puberty disorders, and infertility in men and women caused by hypogonadism (Corona et al., 2011; Skałba and Guz, 2011; Kleine and Rossmanith, 2013; Basaria, 2014; Beneke et al., 2015; Klein et al., 2017). This is often accompanied by mental stress for those affected. Pediatric indications also need to be considered, as many steroid disorders of the adrenal cortex first arise in childhood (Salonia et al., 2019; Yadav and Sharma, 2023). In addition to providing diagnostic results, steroid hormone levels are also measured in serum during fertilization and treatment monitoring (Aubard et al., 1997; Gleicher et al., 2000; Strawn et al., 2000; Zitzmann and Nieschlag, 2000; Diemer et al., 2016; Thomsen et al., 2018; Barbonetti et al., 2020; Armeni et al., 2021). Furthermore, elevated hormone levels in serum can be caused by hormone-producing tumors, both in the adrenal cortex and the gonads (Kleine and Rossmanith, 2013; Beneke et al., 2015).

The high biological variability in hormone levels, caused, for example, by circadian rhythms, individual daily variability, temporary stressors, and the menstrual cycle (Beneke et al., 2015), makes the accurate and reliable determination of hormone levels even more important for diagnostic purposes and treatment monitoring. Gas chromatography (GC) or liquid chromatography (LC) coupled mass spectrometry (MS) is the most reliable method to quantify hormones and is thus considered the “gold” standard (Krone et al., 2010; Stanczyk and Clarke, 2010; Conklin and Knezevic, 2020). However, the procedure is both costly and time-consuming and requires a highly qualified laboratory staff. Therefore, immunoassays are currently still the primary method used for routine clinical measurements. However, previous studies have found discrepancies in the measured serum concentrations of sex hormones between the different immunoassays and in relation to the MS-based reference results (Holst et al., 2004; Wang et al., 2004; Coucke et al., 2007; Soldin and Soldin, 2009; French, 2013; Schofield et al., 2017; Zhou et al., 2017). These discrepancies in immunoassay results indicate differences in the specificities of the antibodies used or inappropriate tracers in the competitive assay formats as well as a lack of standardization of the measurement methods. Efforts to standardize immunoassay methods with respect to MS-based reference methods have been underway for many years (Vesper et al., 2008; Vesper and Botelho, 2010; Vesper and Botelho, 2012; Greaves et al., 2016). Moreover, certified reference materials (CRM) for testosterone, progesterone and 17β-estradiol measurements have been existing for several years (Koumantakis, 2008; Zhou et al., 2017; NIST, updated 2020) and can be used to standardize the respective immunoassays.

This study examines whether these standardization efforts have led to an improvement in testosterone, progesterone and 17β-estradiol immunoassay analytics in recent years. The analysis is based on the manufacturer-specific results of an external quality assessment (EQA) scheme conducted by INSTAND - Society for Promoting Quality in Medical Laboratories e.V. between early 2020 and May 2022.

## 2 Materials and methods

### 2.1 Sample materials–preparation and properties

In each EQA survey, two serum samples with different concentrations of testosterone, progesterone and 17β-estradiol were distributed to the participating laboratories for quantitative analysis. The specific analyte concentrations were obtained by spiking pooled human sera with synthetic steroid hormones. The material was stabilized with 0.02% sodium azide and sampled in 2 mL aliquots. The stability and homogeneity of the EQA samples were in line with DIN EN ISO/IEC 17043:2010. The liquid samples were stored at −18°C until they were dispatched to participants at ambient temperature.

### 2.2 Reference measurement procedure

Reference measurement procedures (RMP) are internationally recognized analytical methods of the highest metrological order, making the reference measurement value (RMV) ideally qualified as a target value for the evaluation of laboratory performances in external quality controls. The RMVs for testosterone, progesterone and 17β-estradiol were determined by the INSTAND calibration laboratory, which is accredited according to DIN EN ISO/IEC 17025:2018 and DIN EN ISO/IEC 15195:2019. As established RMP for the three steroid hormones, isotope dilution GC-MS (GC-ID/MS) was used. Metrological traceability was established using primary reference standards (Testosterone NMIJ CRM 6002-a, progesterone NMIJ CRM 6003-a, 17β-estradiol NMIJ CRM 6004-a). In order to assign testosterone values, samples were spiked gravimetrically with ^1^³C₂-testosterone as the internal standard and equilibrated, then precipitated with aqueous KOH, centrifuged, and the supernatant was extracted into dichloromethane. Derivatization was performed with cyclohexane-HFBA and subsequently extracted into cyclohexane phase. GC-MS measurements were done at m/z 680 and m/z 682 (Thienpont et al., 1994). For progesterone measurements, samples were spiked gravimetrically with ^1^³C₂-progesterone as the internal standard and equilibrated, then extracted into n-hexane. This was followed by centrifugation and evaporation of the supernatant to dryness. Derivatization was performed with HFBA in cyclohexane. GC-MS measurements were done at m/z 510 and m/z 512. In order to assign target values for 17β-estradiol, the samples were spiked gravimetrically with ^1^³C₂-estradiol as the internal standard, equilibrated, then extracted into dichloromethane, followed by a clean-up step with Sephadex LH-20. Derivatization was performed with cyclohexane/acetone/HFBA. The GC-MS measurements were done at m/z 664 and m/z 666 (Siekmann, 1984). Six measurements were performed for each target value (two measurements per day on three consecutive days). Measurement uncertainty was assigned to each target value on the basis of a measurement uncertainty budget.

### 2.3 EQA procedure

The INSTAND EQA scheme for measuring testosterone, progesterone and 17β-estradiol is conducted worldwide six times a year (surveys T1 to T6). Two serum samples with two different concentrations (see Section 2.1.) are used per survey (samples S1 and S2). The participating laboratories determine concentrations of testosterone, progesterone, and 17β-estradiol and report on their results via the platform RV-Online (http://rv-online.instandev.de). In addition to submitting the quantitative results for the three steroid hormones, participants are to provide INSTAND with information on the respective device, reagent and method used.

As an RMP is available for testosterone, progesterone and 17β-estradiol, the RMV served as the target value for the evaluation of the EQA results, regardless of the test assays or devices used by the laboratories. For all three steroid hormones, the EQA passing criterion for certification was a deviation from the target value of no more than ±35% according to the rules set out in the guideline of the German Medical Association for quality assurance of medical laboratory analyses (Rili-BÄK) (Bundesärztekammer, 2023).

### 2.4 Data analysis and statistics

The EQA results for testosterone, progesterone and 17β-estradiol were analyzed for the manufacturer collectives for surveys 2020-T1 to 2022-T3. The number of reported results were generally low for the T2 surveys, making a manufacturer-specific analysis statistically less meaningful. Therefore, only the five other surveys (T1, T3 - T6) were considered in this study. Accordingly, the raw data of twelve surveys in total were analyzed.

Values that scattered farther than 4-fold the standard deviation (SD) of the various collectives were defined as outliers and excluded from the statistical analysis. This definition of outliers primarily excludes gross errors from the analysis that are most likely due to a sample mix-up or a reporting error by individual participants. Thus, ten testosterone results, fourteen progesterone results, and thirteen 17β-estradiol results were excluded (for raw data see Supplementary Table S1).

For all three analytes, the test manufacturer collectives with the highest number of participants per survey were considered, i.e., Abbott (AB), bioMérieux (AX), Siemens and Roche (RO). Siemens consisted of five sub-collectives that showed discrepant results. Therefore, the Bayer Healthcare (SI (BG)) and DPC Biermann (SI (DG)) collectives were presented separately in the analyses. The Dade Behring (SI (BW)), the Siemens Healthineers (SIE) and the Siemens Medical Solutions Diagnostics (SI) collectives had only sporadic participants and were excluded from the analyses. In the case of testosterone, the rather small Tosoh Bioscience (TH) collective was also included as the number of participants increased over the period under observation. See the raw data for details on the assays and devices used by the participating laboratories (Supplementary Table S1).

The distribution of the manufacturer-specific inter-laboratory results for testosterone, progesterone and 17β-estradiol were presented longitudinally as boxplot diagrams. The whiskers of the boxes were defined to stretch from the first quartile −1.5 × (interquartile range) to the third quartile +1.5 × (interquartile range). Further statistical information is provided in Supplementary Table S2. As an RMP is available for all three analytes, the assay-dependent deviations from the RMV were calculated for the EQA results and normalized to the RMV, hereafter designated as bias. The distributions of the bias results for testosterone, progesterone and 17β-estradiol were visualized as boxplot diagrams for sample 2. The normalized manufacturer-dependent biases were examined in relation to the EQA evaluation criterion of ±35% for all three steroid hormones in accordance with the Rili-BÄK guideline (Bundesärztekammer, 2023).

The distribution of the absolute EQA results for the three steroid hormones is provided in the (Supplementary Figure S1).

The EQA results were correlated with the RMV in order to check whether the relative bias of individual manufacturer collectives might indicate a concentration dependency. The manufacturer-specific regression lines could be compared with the y (RMV) = RMV reference line as well as the lower and upper EQA limit of ±35%.

In order to obtain an impression of the value scatter within the individual manufacturer collectives, the coefficients of variation (CV) were calculated for all three steroid hormones.

Basic statistical analyses were performed using JMP 17.0.0 from SAS Institute (Cary, North Carolina, United States).

### 2.5 Image generation

The overlay images were generated using the Gnu image manipulator software 2.10.8.

## 3 Results

This study evaluates the quality of inter-laboratory measurements of testosterone, progesterone and 17β-estradiol conducted between early 2020 and May 2022. In a total of twelve EQA surveys, 2,972 results for testosterone, 2,146 for progesterone and 2,292 for 17β-estradiol were reported by 280 participating laboratories (Supplementary Table S1). After selecting the collectives and eliminating outliers (see Section 2.4.), 2,314 results for testosterone, 1,743 results for progesterone and 1,904 results for 17β-estradiol from 128 laboratories were presented graphically (Supplementary Table S2).

**FIGURE 1:**
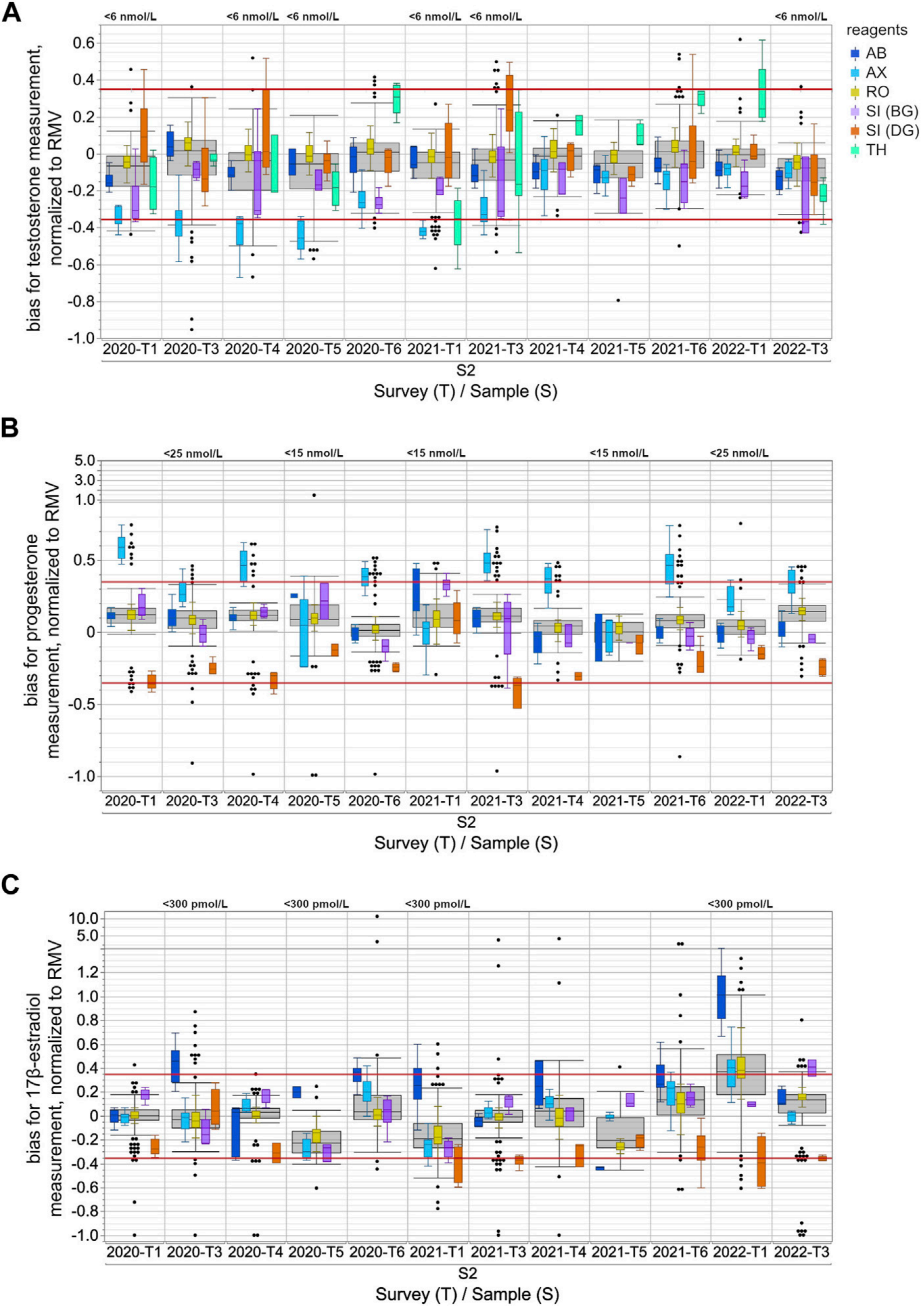
Assay-dependent EQA data for testosterone **(A)**, progesterone **(B)** and 17β-estradiol **(C)** measurements in human sera from 2020-T1 to 2022-T3, normalized to the respective reference measurement value (RMV). Only the results for the S2 samples are shown and are representative of all samples. The surveys with EQA samples with low concentrations, testosterone level <6 nmol/L, progesterone level <15 nmol/L or <25 nmol/L, and 17β-estradiol level <300 pmol/L, are labeled in the upper part of the boxplot diagram. Total data is shown as a grey box for the respective survey. The colored boxes show the manufacturer-specific EQA results. The horizontal red line represents the EQA criterion of ±35% of the target value, as determined by reference measurement procedure. For all boxes, the whiskers stretch from the first quartile −1.5 × (interquartile range) to the third quartile +1.5 × (interquartile range). Values outside of this range are shown as dots, but only for the overall results.

High variation within the manufacturer collectives was found for the three steroid hormones throughout the period analyzed. The whisker ranges reveal that the results of the different collectives do not overlap for some EQA samples (Supplementary Figure S1). While the individual manufacturer collectives showed a clear trend towards increased or decreased levels compared to the overall results for testosterone and progesterone detection, there was a concentration-dependent bias for 17β-estradiol determination (Figure 1C, Supplementary Figure S1C).

When normalizing the results of the individual EQA surveys to the RMV, the overall results for testosterone showed a slight tendency towards underestimation, while for progesterone there was a slight tendency towards overestimation (Figure 1). These tendencies seemed to be partly caused by the deviation of the AX collective, which often exceeded the EQA limit of ±35% of the RMV.

**FIGURE 2:**
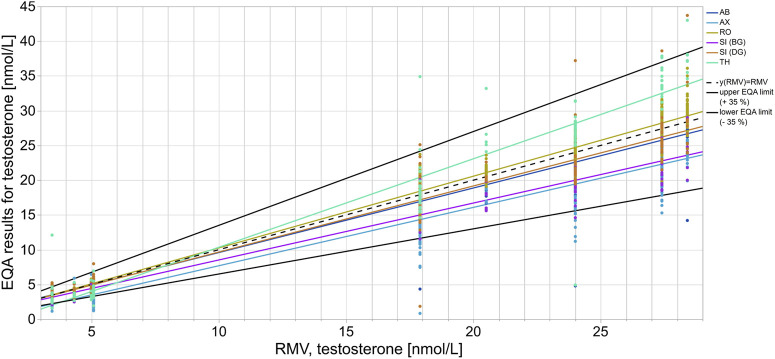
Assay-dependent EQA data as represented here by testosterone quantitation correlated to the reference measurement value (RMV). Each color shows the EQA results of a specific assay collective with the respective regression line. The y (RMV) = RMV correlation line is shown as a reference line (black dashes). The solid black lines represent the accepted EQA criterion of ±35%.

In the case of testosterone, the median of the AX collective consistently showed clear deviations from the RMV of −19.7% to −52.2% for all EQA samples up until 2020-T6 (Figure 1A). After 2021-T6, the median of the AX collective deviated less from the RMV for most EQA samples and was even consistently less than −25%. The SI (BG) collectives showed a lower median than the RMV, with a bias down to −36.6% for several EQA surveys. The median bias of the TH collective varied between −35.0% and +32.4%. Interestingly, the upward deviations were only observed in samples with testosterone concentrations above 20 nmol/L (Supplementary Figure S1A). For samples with lower concentrations, the median bias of the TH collective tended to be negative. A correlation of the inter-laboratory test results with the RMV and a comparison of the manufacturer-specific regression lines with the y (RMV) = RMV identity line confirmed that the bias of the TH collective was concentration dependent (Figure 2). A slighter concentration dependency could also be assumed for the AX collective when the regression line was compared with the −35% EQA limit, since a higher percentage deviation was found for low-concentration testosterone samples than for high-concentration ones.

For progesterone, the median bias of the AX collective was often observed to be above the +35% EQA criterion and even up to +58.9% for sample S2 in 2020-T1 (Figure 1B). In individual EQA surveys, the SI (DG) collective median was also slightly below the −35% EQA criterion.

In the case of 17β-estradiol, the overall results showed the highest upward and downward median bias compared to the median bias for testosterone and progesterone measurement (Figure 1C). Upward deviations of the median of the AB collective were observed for 17β-estradiol concentrations below 600 pmol/L, while for higher concentrations, the results were either closer to the RMW or showed a downward deviation. The results of the SI (BG) collective were remarkably high in the case of 17β-estradiol concentrations above 1,000 pmol/L (Supplementary Figure S1C). In contrast, the medians of the SI (DG) collective were consistently low for all EQA samples regardless of the concentration. However, it should be noted that, over the analyzed period, there was a trend towards more negative deviations in the medians of the SI (DG) collective. Since the beginning of 2021, participants of the SI (DG) collective often struggled to meet the −35% EQA criterion (Figure 1C).

**FIGURE 3:**
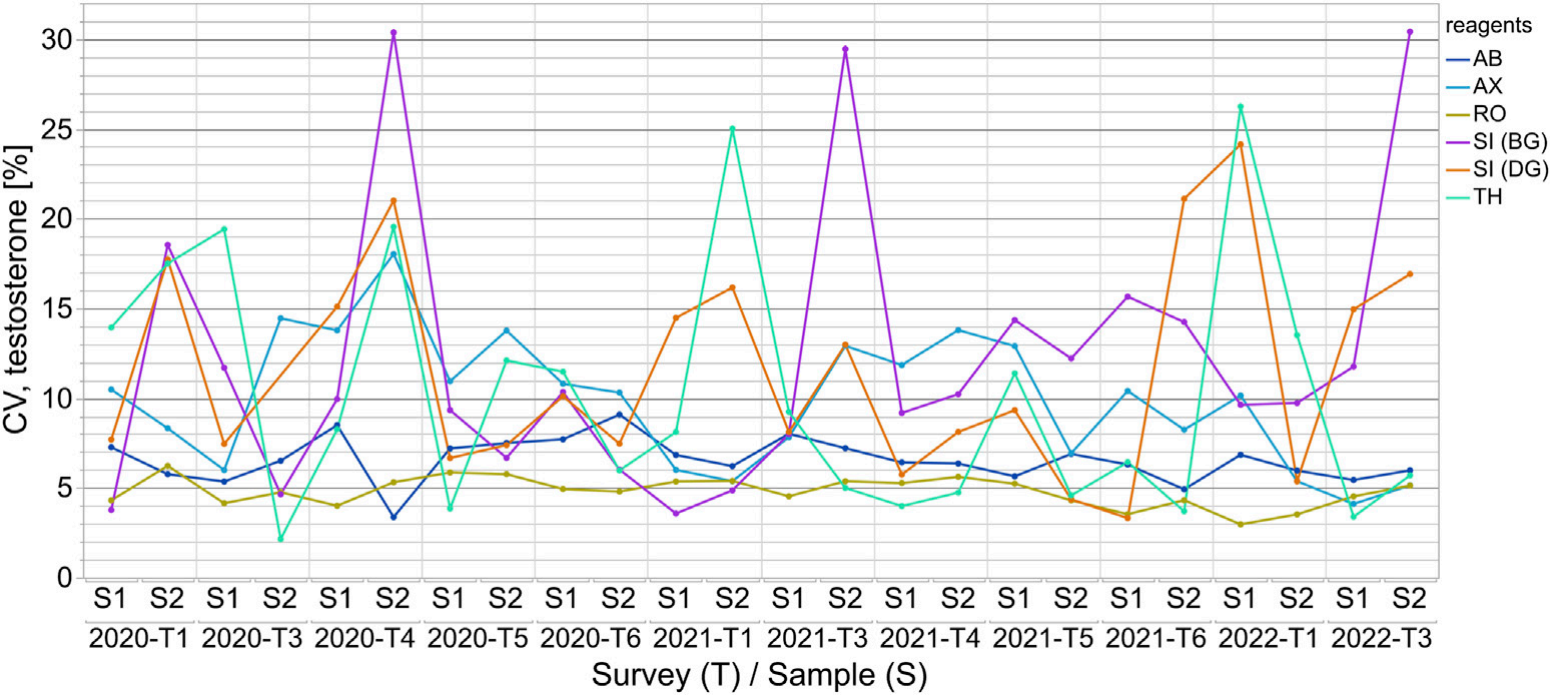
The coefficients of variation (CVs) for the assay-dependent EQA results for testosterone measurements from 2020-T1 to 2022-T3 are shown for samples S1 and S2 for each survey. The results of the surveys are independent of one another and thus the CVs are only linked longitudinally to better visualize the changes over time.

For quantitation of all three steroid hormones, the outlier-adjusted CVs were below 25% with a few exceptions for some manufacturer collectives (Figure 3 and Supplementary Figure S3). In the case of testosterone measurement, the CVs were consistently below 10% for the AB and RO collectives. This also applied to the RO collective for progesterone measurement. CVs were consistently below 15% for the AX and RO collectives for 17β-estradiol measurement. Individual cases of remarkably high CVs were observed for various test collectives for all three sex hormones, however these reached a maximum value of 45% (see Supplementary Figure S3B).

## 4 Discussion

Considering the number of health impairments linked to hormonal disorders (Beneke et al., 2015), reliable and accurate hormone quantitation is essential in order to provide patients with accurate diagnoses and treatment monitoring. However, publications have been reporting for years on the insufficient level of standardization of immunoassays for steroid hormone analysis (Vesper et al., 2008; Vesper and Botelho, 2010; Vesper and Botelho, 2012; Greaves et al., 2016). Certified reference materials are available (Zhou et al., 2017; NIST, updated 2020), but most of the test kit manuals do not provide any information about the traceability of the applied standard samples used to create the respective standard curve or used for 1- or 2-point recalibration. In addition, manufacturers rarely include comparative data with the results of MS-coupled procedures, which are considered the “gold” standard (Krone et al., 2010; Stanczyk and Clarke, 2010; Conklin and Knezevic, 2020). Even though the lack of specificity and selectivity of immunoassays and their disadvantages compared to GC- or LC-MS procedures are well known (Wang et al., 2004; Shackleton, 2010; French, 2013; French, 2016), they are currently still the method of choice in routine measurement as they are practical to carry out and have a high throughput rate. The number of laboratories participating in the EQAs that use MS-coupled methods for steroid hormone determinations has increased in recent years but remains below 10%: around 3% of all 17β-estradiol results, 7% of all testosterone results, and around 8% of all progesterone results (see the raw data in Supplementary Table S1).

This study investigates the quantitative EQA results for testosterone, progesterone, and 17β-estradiol in human serum from twelve INSTAND surveys conducted between early 2020 and May 2022.

The immunoassay-specific results for all three steroid hormones still showed considerable differences. For some EQAs, there was no overlap in the results of different manufacturer collectives when values exceeding the whisker range were disregarded (Figure 1 and Supplementary Figure S1). The EQA results of individual collectives distinctly stood out for progesterone, whereby the overall results of a particular sample overlapped considerably with those of another sample that was twice as concentrated. This was observed with the S2 sample in 2021-T6 and the S1 sample in 2022-T1 (Supplementary Figure S1B).

Normalizing the testosterone, progesterone and 17β-estradiol levels to the RMV allows a comparison to be made of the accuracy of the different immunoassays, even across the several EQA samples and surveys. The median bias of the different collectives was up to approximately 50% for the measurement of both testosterone and 17β-estradiol, and almost 60% for the determination of progesterone (Figure 1). In the case of the 17β-estradiol measurement, the S2 sample in 2022-T1 proved to be an exception with a considerably higher percentage deviation between the manufacturer-dependent results. While both Siemens sub-collectives had similar median biases compared to the other EQA samples, the other three collectives showed substantially higher upward deviations. One can assume that a cross-reacting compound in this particular sample interfered with the measurement of 17β-estradiol in the AX, RO, and especially the AB immunoassays (Sturgeon and Viljoen, 2011; Wauthier et al., 2022), however the compound did not interfere with the measurement of testosterone or progesterone (Figure 1). An interfering substance in an EQA sample may be either of endogenous origin in the serum matrix or due to artificial additives which are used during sample preparation for the purpose of stabilization or spiking. Since the manufacturing process of the EQA sample remained the same for all of the analyzed EQA surveys, it can be assumed this was not caused by an artificial additive in this sample. The fact that test kits from other manufacturers were not impacted by this presumably interfering compound shows that the immunoassays in these kits may be more effectively protected against cross-reacting substances than the methods mentioned above.

The high structural and steric similarity of the numerous derivates in the steroid family means that differentiation by antibody detection is difficult due to cross-reactivity (Krasowski et al., 2014; Yamamoto et al., 2014; Beneke et al., 2015) and thus poses a major challenge for the immunoassay measurement of steroid hormones. Test manufacturers list several cross-reacting molecules in their test manuals, e.g., in progesterone analyses, the rate of a cross-reaction with 11-deoxycorticosterone is 1%–4% depending on the test. In the test manuals for testosterone measurement, much higher interference rates of up to 34% are reported for 11β-hydroxy-testosterone and 11-keto-testosterone. Krasowski et al. found higher cross-reactivities for testosterone measurement than for progesterone and 17β-estradiol determination in the Roche Diagnostics Elecsys assays (Krasowski et al., 2014).

The many possible interfering substances can lead to both an over- and underestimation of testosterone, progesterone and 17β-estradiol levels (Sturgeon and Viljoen, 2011). In general, overestimated steroid hormone levels in serum can result in the erroneous diagnosis of hormonal diseases and cause avoidable uncertainty among patients. Underestimated sex hormone levels can falsely lead to a presumed case of hypogonadism and, in turn, unnecessary hormone substitution in patients (Zitzmann and Nieschlag, 2000; Zitzmann et al., 2006). To avoid misdiagnoses, hormone measurements should be interpreted with caution, especially for patients on medication, since cross-reactivity occurs with drugs that have a high structural similarity, e.g., with methyltestosterone in some testosterone immunoassays (Krasowski et al., 2014).

As a consequence, the same immunoassay should be used for patient monitoring and follow-up in order to minimize discrepant results and uncertainty for clinicians and patients due to possible assay-dependent under- or overdetermination in steroid hormone measurement.

Most manufacturer collectives deviated either upwards or downwards from the RMV when quantifying steroid hormones, however some collectives showed deviations from the RMV in both directions (Figure 1). In the case of 17β-estradiol quantitation, positive as well as negative biases to the RMV were observed for all manufacturer collectives, as well as for the total collective. In these cases, the deviations of the assay collective seemed to depend on the hormone concentration in the EQA sample (Figure 2, Supplementary Figures S1, S2).

The testosterone results for the TH collective were remarkably higher than the RMV for samples with high concentrations, e.g., sample S2 in 2020-T6, 2021-T4, 2021-T6 and 2022-T1. In contrast, samples with concentrations below 6 nmol/L were underestimated, see sample S2 in 2020-T1, 2020-T4, 2020-T5, 2021-T1, 2021-T3 and 2022-T3 (Figure 1 and Supplementary Figure S1). This concentration dependency might be due to an imprecise test calibration or due to insufficient sensitivity in cases of low steroid hormone concentrations. However, the testosterone concentrations of the EQA samples were within the measuring ranges specified in the test manuals of the assay manufacturers and were within clinically relevant concentrations (Beneke et al., 2015).

Kanakis and others found that most commercially available immunoassays used for testosterone quantitation are insufficient for lower concentrations within the normal reference range for men (∼10 nmol/L to ∼35 nmol/L) and the entire reference range for women (∼0.2 nmol/L to ∼3 nmol/L). For this reason, slight androgen excess in female patients cannot be measured by some of the commercial tests and remains undetected (Kanakis et al., 2019). This problem is addressed, for example, in EQA samples S1 in 2020-T3 and S2 in 2021-T3 representing elevated female serum testosterone levels. These elevated levels would likely not be identified using the AX or the TH immunoassays due to underestimation (Supplementary Figure S1A). This can result in an unreliable diagnosis of diseases associated with androgen excess in women, such as idiopathic hirsutism, PCOS, hyperthecosis ovarii, late-onset congenital adrenal hyperplasia or testosterone-producing tumors. Some groups reported challenges in measuring low serum testosterone concentrations (La’ulu et al., 2018; Cao et al., 2019; Kanakis et al., 2019). La’ulu et al. described sensitivities for testosterone measurement with various commercial immunoassays in concentrations ranging from 0.36 nmol/L to 3.49 nmol/L (Schwartz et al., 1986; Legro et al., 2013; Beneke et al., 2015; Azziz, 2018; Cussen et al., 2022). On the other hand, samples with low levels of steroid hormones can also be overestimated, as interfering substances and cross-reactivities could overwhelm the measurement of the target analyte. This would result in unrecognized hypogonadism in patients (Corona et al., 2011; Skałba and Guz, 2011; Basaria, 2014; Beneke et al., 2015; Klein et al., 2017).

The same challenges arise when measuring low concentrations of progesterone (<5 nmol/L) and 17β-estradiol (<40.7 pmol/L) (Oettel and Mukhopadhyay, 2004; Huhtaniemi et al., 2012; Shankara-Narayana et al., 2016) in male patients or in women with depressed levels. For EQA result distribution for EQA samples with progesterone concentrations <5 nmol/L see also sample S2 in 2020-T5 and 2021-T2 (Figure 1). For 17β-estradiol, the lowest concentrations in the EQA scheme were around 150 pmol/L, e.g., sample S2 in 2021-T1 and sample S1 in 2021-T5. The EQA results reveal clear measurement differences between the individual collectives (Figure 1 and Supplementary Figure S1). All in all, an improvement in immunoassay measurements is especially desirable for samples with low hormone levels and should be pursued further by the current standardization programs.

While the wide variations within the manufacturer collectives in testosterone, progesterone and 17β-estradiol immunoassay measurement revealed issues with accuracy, within-assay agreement was mainly good, indicating relatively good analytical precision. The outlier-adjusted scatter within the collectives was found to be mostly inconspicuous (Figure 3, Supplementary Figure S3) and similar to the manufacturer’s specifications in the test manuals. The CVs for the manufacturer collectives were, with few exceptions, below 15% for all three steroid hormones. For testosterone quantitation, slightly higher CVs were observed for the SI (BG), SI (DG) and TH collectives than for the others. This was most likely due to the lower number of EQA results for these collectives. In the case of progesterone and 17β-estradiol determination, the CVs for SI (DG) and SI (BG), and, in the case of 17β-estradiol, for the AB collective as well, should be interpreted with caution for the same reason. For all three hormones and all test collectives, slightly increased CVs were observed over two to three consecutive surveys. One possible explanation for this could be lot changes by manufacturers.

Overall, the bias analysis of the testosterone, progesterone and 17β-estradiol data confirmed the findings of previously published studies, which found that immunoassays were insufficiently reliable in quantitatively determining sex hormones. A trend towards standardizing immunoassay detection for steroids has yet to be observed (Vesper et al., 2014; Lawrenz et al., 2018). However, this EQA data revealed one exception. The dispersion of testosterone values between the different assays decreased over the studied period. This can be ascribed to the development towards a higher accuracy in the AX collective. Until 2021-T3, the results of the AX collective had often exceeded the EQA criterion of −35%. Since the beginning of 2021, the median of the AX collective has remarkably moved closer to the RMV (Figure 1A). This improvement in accuracy could be due to a successful recalibration by the manufacturer. Test system recalibrations have to be performed under consideration of traceability (Koumantakis, 2008). The increased accuracy in testosterone quantitation for the AX immunoassay since 2021 is a good example of how external quality control schemes can reveal inadequate test performance, a matter which can subsequently be discussed with the manufacturers. This can ultimately help improve analytics and thus promote quality assurance in medical laboratories.

One limitation of this study is that stabilized and spiked serum samples were used for the EQAs. However, since manufacturer-dependent deviations in steroid hormone measurements are also described for fresh serum samples in other studies (Taieb et al., 2003; Wang et al., 2004; Coucke et al., 2007; Bell et al., 2012; Cao et al., 2019), it is rather unlikely that the observed manufacturer-specific deviations in the EQA results are primarily due to insufficient commutability of the EQA samples. To make sure that the manufacturer-dependent deviations from the RMV were not, or only negligibly, influenced by the artificial nature of the samples, INSTAND will address this aspect in detail in further studies by providing fresh, non-processed serum samples.

## 5 Conclusion

While the scatter within the manufacturer collectives of the EQA was not critical for the quantitation of testosterone, progesterone and 17β-estradiol using immunoassays, there were considerable differences between the manufacturer-specific EQA results. This revealed the need for distinct improvement in standardization. The increased accuracy of the AX immunoassay in measuring testosterone in the INSTAND EQAs between 2020 and 2022 might be due to successful recalibration of the assay and raises hope for further improvement in the standardization of immunoassays for steroid hormone analysis in the coming years.

## Data Availability

The original contributions presented in the study are included in the article/Supplementary Material, further inquiries can be directed to the corresponding author.
