# Supplementary material for: Longitudinal analysis of external quality assessment of immunoassay-based steroid hormone measurement indicates potential for improvement in standardization
Source: Front Mol Biosci. 2024 Jan 31;11:1345356. doi: 10.3389/fmolb.2024.1345356 (PMC10865096; doi:10.3389/fmolb.2024.1345356)
Supplement: Supplementary file 2 [file DataSheet1.PDF]

Supplementary Figures

1. Supplementary Figures

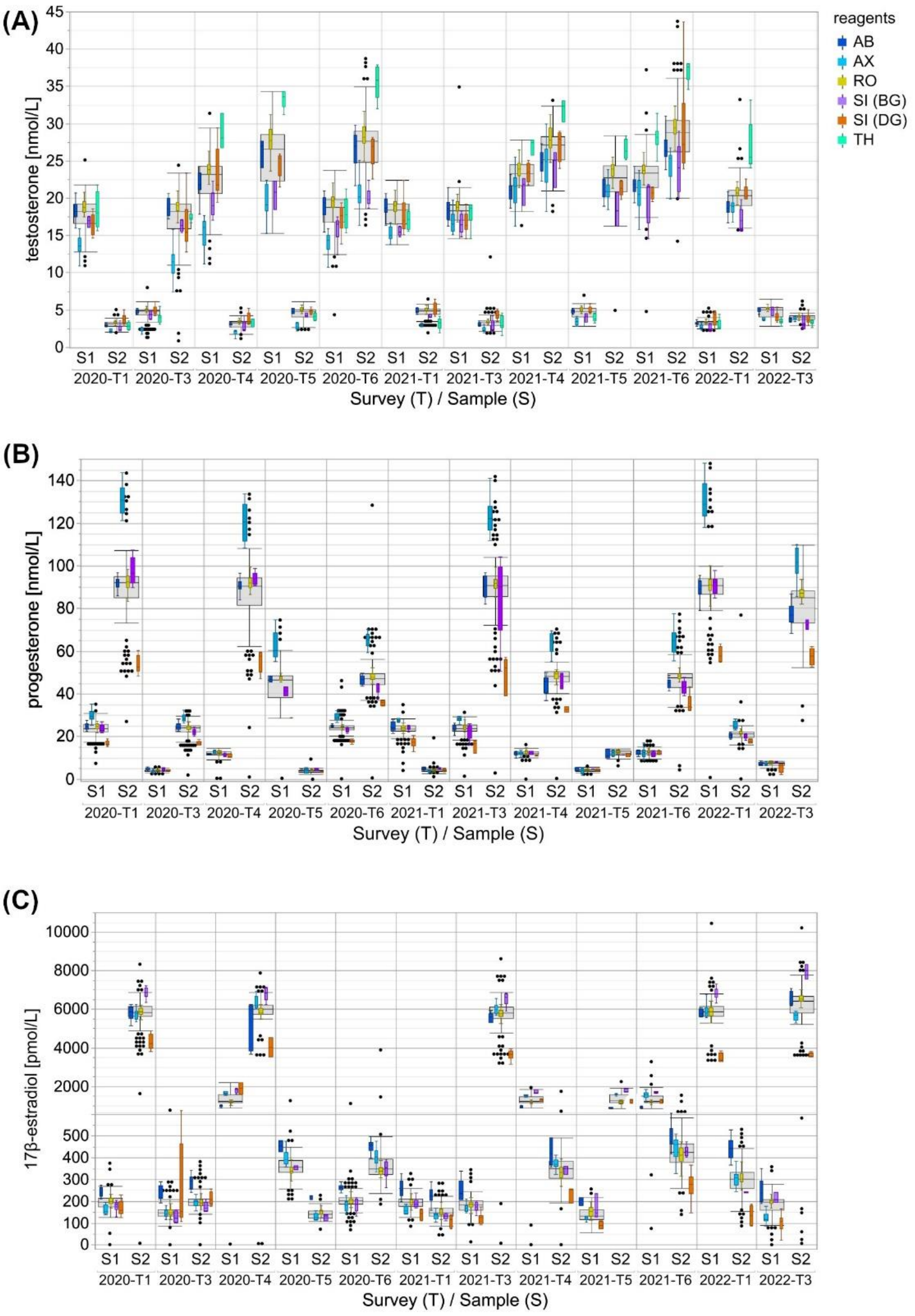

Supplementary Figure S1: Longitudinal assay-dependent analysis of EQA results for testosterone (A), progesterone (B) and 17β-estradiol (C) measurements in human sera from 2020 to 2022. EQA results are shown for five of the six surveys (T) per year, with the second survey of the year (T2) excluded due to low participant numbers. Participants determined steroid hormone concentrations in two samples (S1 and S2) per survey. Total data is shown as a grey box for the respective survey. The colored boxes show the manufacturer-specific EQA results. For all boxes, the whiskers stretch from the 1st quartile − 1.5 × (interquartile range) to the 3rd quartile + 1.5 × (interquartile range). Values outside of this range are shown as dots, but only for the overall results.

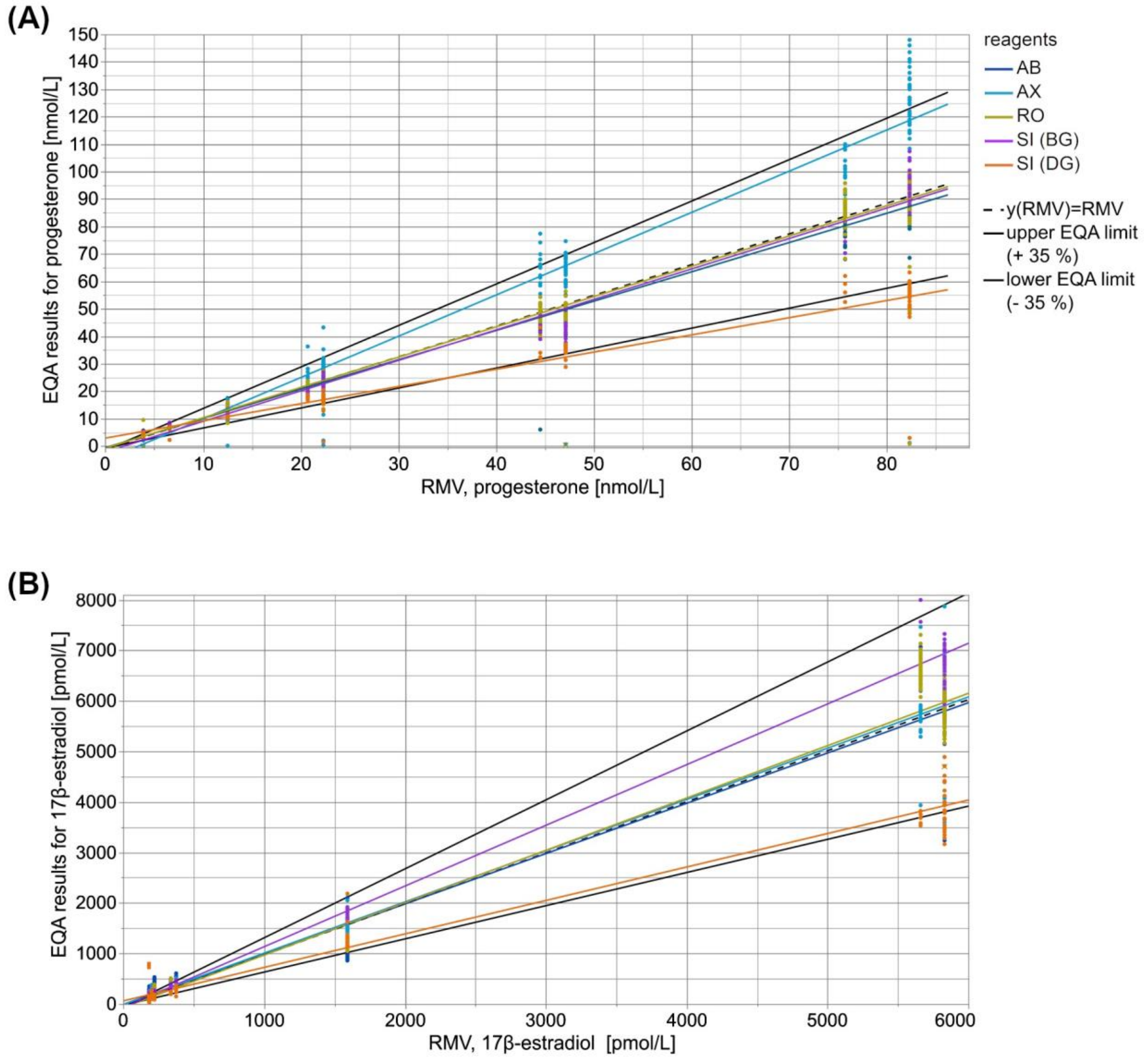

Supplementary Figure S2: Assay-dependent EQA data for progesterone (A) and 17β-estradiol (B) quantitation correlated to the reference measurement value (RMV). Each color shows the EQA results of a specific assay collective with the respective regression line. The y(RMV)=RMV correlation line is shown as a reference line (black dashes). The solid black lines represent the accepted EQA criterion of ± 35 %.

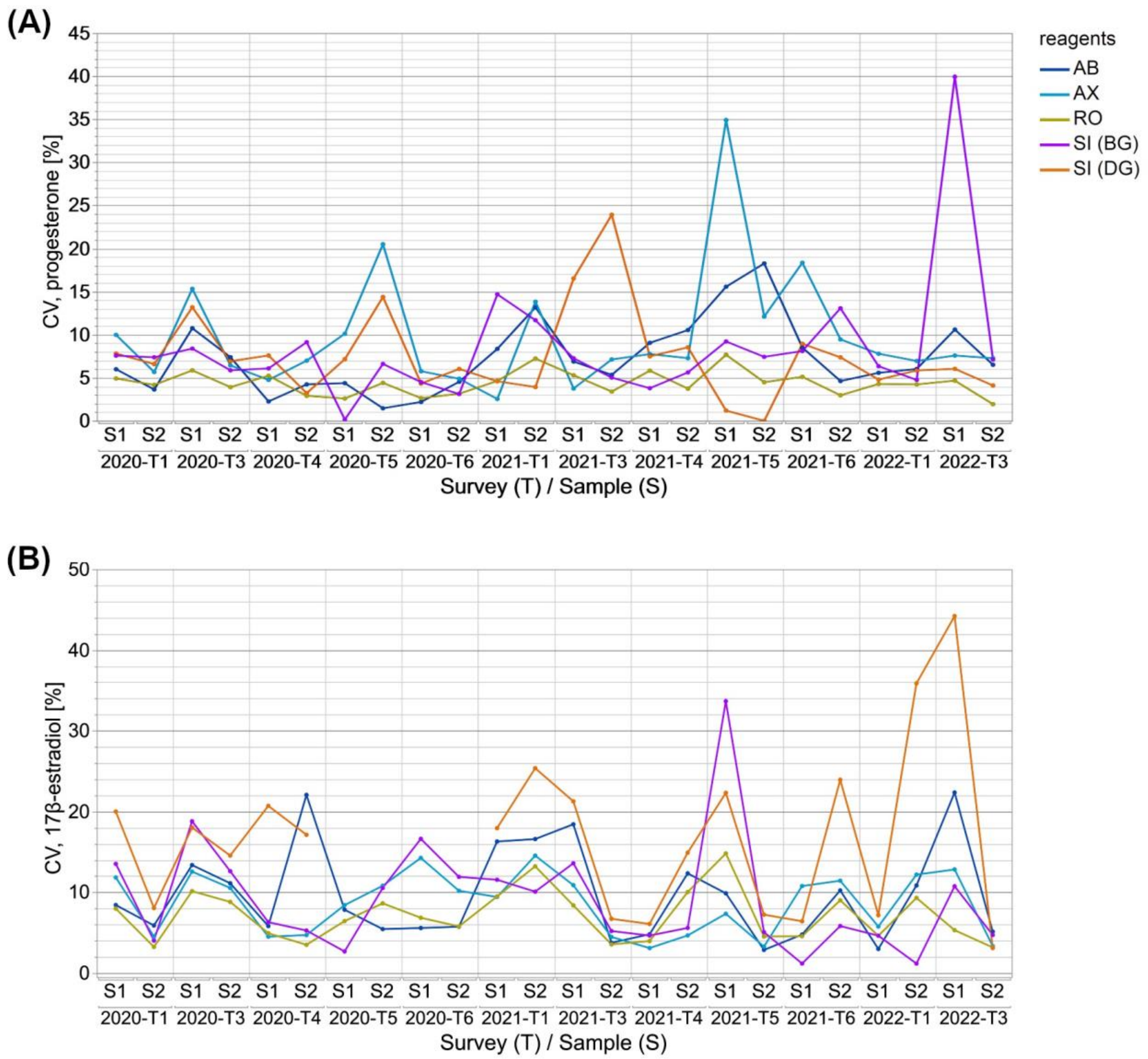

Supplementary Figure S3: The coefficients of variation (CVs) for the EQA results for progesterone (A) and 17β-estradiol (B) measurements from 2020-T1 to 2022-T3 are shown for samples S1 and S2 for each survey. The results of the surveys are independent of one another and thus the CVs are only linked longitudinally to better visualize the changes over time.
